# Using Wearable Digital Devices to Screen Children for Mental Health Conditions: Ethical Promises and Challenges

**DOI:** 10.3390/s24103214

**Published:** 2024-05-18

**Authors:** Aisling O’Leary, Timothy Lahey, Juniper Lovato, Bryn Loftness, Antranig Douglas, Joseph Skelton, Jenna G. Cohen, William E. Copeland, Ryan S. McGinnis, Ellen W. McGinnis

**Affiliations:** 1 Department of Philosophy, Virginia Polytechnic Institute and State University, Blacksburg, VA 24060, USA; afoleary@vt.edu; 2 University of Vermont Medical Center, Burlington, VT 05401, USA; timothy.lahey@uvmhealth.org (T.L.); antranig.douglas@uvm.edu (A.D.); 3 Complex Systems Center, University of Vermont, Burlington VT 05405, USA; juniper.lovato@uvm.edu (J.L.); bryn.loftness@uvm.edu (B.L.); 4 Department of Pediatrics, Wake Forest University School of Medicine, Winston-Salem 27101, NC, USA; jskelton@wakehealth.edu; 5 Division of Public Health Sciences, Wake Forest University School of Medicine, Winston-Salem 27101, NC, USA; 6 Department of Electrical and Biomedical Engineering, University of Vermont, Burlington VT 05405, USA; jenna.g.cohen@uvm.edu; 7 Department of Psychiatry, University of Vermont, Burlington VT 05405, USA; william.copeland@med.uvm.edu; 8 Department of Biomedical Engineering, Wake Forest University School of Medicine, Winston-Salem 27101, NC, USA

**Keywords:** mental health, pediatrics, digital health, ethics, artificial intelligence, wearables

## Abstract

In response to a burgeoning pediatric mental health epidemic, recent guidelines have instructed pediatricians to regularly screen their patients for mental health disorders with consistency and standardization. Yet, gold-standard screening surveys to evaluate mental health problems in children typically rely solely on reports given by caregivers, who tend to unintentionally under-report, and in some cases over-report, child symptomology. Digital phenotype screening tools (DPSTs), currently being developed in research settings, may help overcome reporting bias by providing objective measures of physiology and behavior to supplement child mental health screening. Prior to their implementation in pediatric practice, however, the ethical dimensions of DPSTs should be explored. Herein, we consider some promises and challenges of DPSTs under three broad categories: accuracy and bias, privacy, and accessibility and implementation. We find that DPSTs have demonstrated accuracy, may eliminate concerns regarding under- and over-reporting, and may be more accessible than gold-standard surveys. However, we also find that if DPSTs are not responsibly developed and deployed, they may be biased, raise privacy concerns, and be cost-prohibitive. To counteract these potential shortcomings, we identify ways to support the responsible and ethical development of DPSTs for clinical practice to improve mental health screening in children.

## 1. Introduction

Only half of the nearly eight million US children with a mental health disorder receive treatment from a mental health professional [1] despite increases in clinically significant anxiety and depression across the globe [2], from preschoolers [3,4] to adolescents [5], after the COVID-19 pandemic. In light of these staggering statistics, the United States Preventive Services Task Force (USPSTF) and the American Academy of Pediatrics (AAP) Task Force on Mental Health recommend that pediatricians screen all children for mental health impairment in hopes of preventing or ameliorating mental health crises [6,7,8].

Screening children for mental illness will require a significant shift in practice for pediatricians. Though pediatricians overwhelmingly agree that they should identify children’s mental health problems [9], most do not use standardized tools to screen children for mental health issues [10]. They cite barriers to screening such as time and scheduling constraints to score and interpret surveys [8], and thus often administer screening surveys only if the caregiver or child reports a mental health concern. This places the onus solely on caregivers to accurately observe, raise concern for, and report on a child’s internal emotional state. However, subjective caregiver-reports of child mental health are biased as the emotional suffering of others is inherently unobservable [11,12], acknowledgement of problems requires mental health literacy [13], and reporting concerns to a provider requires immense trust in health care systems [14] and is often related to a family’s sociodemographic factors [15].

**Figure 1:**
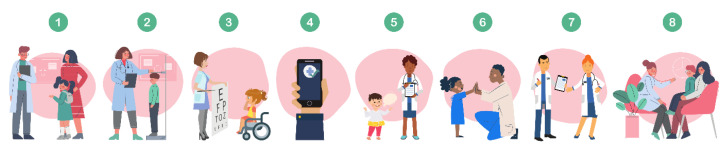
An example of how DPSTs could be deployed in pediatric practice. (1) A child and their guardian arrive for their annual pediatric well-visit. (2) The visit begins with traditional longitudinal screening activities, such as the tracking of height and weight. (3) After being administered an eye exam at their eight-year pediatric well-visit, (4) the child stands next to their mother as a nurse secures a clinic-owned smartphone to the child’s lower back with an elastic waist belt. (5) The nurse opens an app connected to the smartphone and instructs the child to tell a story ‘that will be judged based on how interesting it is.’ The app continuously collects movement and vocal biomarkers during the three-minute task. (6) The nurse thanks the child and tells them ’what a great job’ they did. The app immediately feeds the recorded movement and vocal biomarkers into a machine learning model that reports the likelihood of the child having clinically elevated levels of anxiety or depression. (7) Instantaneously, the data are uploaded to the child’s electronic health record (EHR) along with automated recommendations to the pediatrician for supplemental mental health screening needs, (8) which may include caregiver-report surveys.

**Figure 2:**
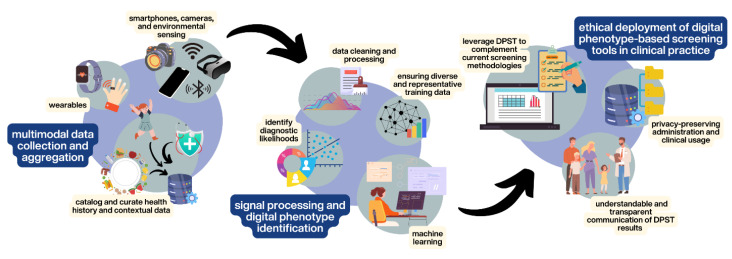
The development lifecycle of DPSTs: (1) Multimodal data are collected and aggregated from digital devices along with contextual health data. (2) Data from diverse training data are processed and machine learning algorithms are leveraged to identify patient diagnostic likelihood and digital phenotypes. (3) Ethical deployment of the DPSTs to supplement current screening methods is tested in clinical practice with consideration for privacy and interpretability of patient data and experiences.

Digital phenotype-based screening tools (DPSTs) may help surmount these obstacles by leveraging sensor data passively collected by smartphones or other wearable devices to screen for mental health disorders (Figure 1). Research studies to date have developed DPSTs (Figure 2) and are in evaluative stages for Attention Deficit Hyperactive Disorder (ADHD) [16,17], bipolar disorder [18], and internalizing disorders [19,20,21,22,23] in children. Our team has found that digital phenotyping can take as little as one to three minutes conducted in a medical office space [21], and others have found that screening could occur remotely between clinical visits [17] which may help overcome time barriers to pediatrician screening for mental illness. In practice, DPSTs could assess a child’s movement, voice, heart rate variability, respirations, eye movements, and galvanic skin response [24,25,26,27,28,29] while they react to a brief potentially threatening situation like walking into a dimly lit room or giving a speech while wearing a small monitor that resembles an electrocardiogram lead or watch-like device [21]. After data collection, devices incorporate artificial intelligence (AI), typically using machine learning techniques to generate diagnostic likelihood [30], which informs the pediatrician delivering feedback to the patient’s family. These advanced data analysis approaches help to reveal the complex relationships that exist between objective physiological and behavioral measurements and underlying mental health conditions. Figure 1 is an example of how DPSTs like this could be deployed in pediatric practice.

**Figure 3:**
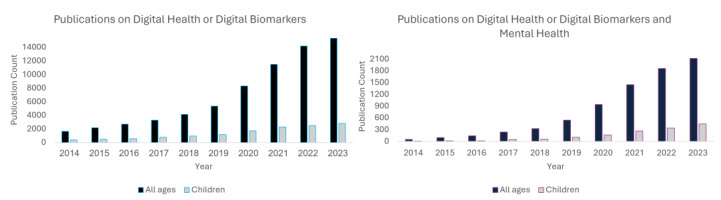
As a proxy for the emerging popularity of DPSTs, publications focusing on digital health or digital biomarkers have shown significant growth in the last decade (**left**), increasing more than 800%. Publications in this area focusing on mental health have shown even more explosive growth (**right**) with an increase of more than 4000% during the same period. Papers focused specifically in pediatric populations represent a small but growing percentage (~20%).

The development of DPSTs has accelerated considerably in recent years as evidenced by increases in scientific publications in the space (more than 800% in the past decade, Figure 3) and funding for digital health companies, which reached more than $23 billion in 2022 [31]. The application of these tools in mental health have shown even more explosive growth with a more than 4000% increase in publications in the last decade. Initial efforts targeting mental health have largely considered their use in adults, leveraging passively collected sensor data from smart phones and other sensors to identify phenotypes of mental health disorders and changes in their associated symptoms (e.g., [32,33,34,35,36,37]). A particular focus has been on vocal biomarkers of mental health [38,39,40,41] which have quickly emerged as one of the most promising and feasible measures to consider. More recent efforts are beginning to expand to consider additional data sources including biomarkers derived from wearable movement sensors [16,17,22], videos of body and facial movements [42,43], and a variety of physiological measurements such as heart rate, heart rate variability, respirations, and galvanic skin response [24,25,26,28,29]. These techniques, as described above, are now starting to move from adults to children [20,21,22,24,25,26,28,29,30,44,45,46]. Notably, a small number of companies are now pursuing digital phenotype diagnostics or diagnostic aids targeted specifically at pediatric mental health. Cognoa is one example, who recently secured FDA clearance for a technology that provides clinician support for diagnosing autism using video-based assessments and AI [47]. Another example is Kiddo Health, which provides a wearable-based connected care platform for monitoring biomarkers in children with physical and behavioral health challenges [48].

The use of DPSTs for mental illness in childhood raises important ethical issues which should be thoroughly examined before routine implementation in pediatric practice. Additionally, these issues may be distinct from ethical implications of digital phenotyping in late adolescence and adulthood. Herein, we examine some of the major ethical ramifications of wearable digital mental health screening for children under three broad categories: accuracy and bias, privacy, and accessibility and implementation.

## 2. Accuracy and Bias

Currently, the gold standard for screening for mental health disorders in children consists of surveys administered to children and their caregivers prior to, or during, pediatric visits [49,50]. Screening surveys for anxiety and depressive disorders in school-age children and adolescents exhibit good accuracy (i.e., as high as 88% for the nine-item Patient Health Questionnaire in screening for depression [51]), but with variable sensitivity and specificity (i.e., as low as 50% and 56%, respectively, for screening for any anxiety disorder [6]), and with positive screens for anxiety and depression ranging from 11% to 15% in representative pediatric outpatient samples (using the Pediatric Symptom Checklist-17 [52]). To supplement caregiver responses, children fill out diagnostic surveys reporting on their own symptoms when they can. Child self-reporting of symptoms typically begins between ages 8 and 11 (e.g., the SCARED survey is designed for children ages 8–18, and the ASEBA Youth Self-Report is intended for children ages 11–17 [53]). Caregiver and youth reporters describe symptoms across multiple contexts, with reports typically exhibiting good ecological validity [54]. The exclusion of very young children from the diagnostic process is understandable; these children often cannot reliably report on their inner thoughts, feelings, and emotions [55,56]. Parent and guardian responses too have limitations since their responses to mental health surveys often conflict with their children’s [57]. Compared to child self-reports, most caregivers report fewer anxiety and depressive symptoms and more behavioral problems [58,59,60,61,62,63]. Conversely, caregivers with mental health impairments themselves have been demonstrated to over-report on their children’s problems [11,12]. Importantly, caregiver and child dyads that are most discordant with each other exhibited decreased follow-up clinical care [64] and unfavorable long-term rates of engagement in school, work, and the criminal justice system [65].

The accuracy of DPSTs for mental health disorders has been more thoroughly studied in adults than in children. Nevertheless, our team has found that digital phenotyping for mental health screening in children demonstrates promising accuracy (i.e., 80–81% [24,44]) and specificity (i.e., 88% [44]) in detecting anxiety and depressive disorders, but variable sensitivity similar to that of screening surveys (e.g., as low as 54% [24]). With that said, some have noted the benefit of having high specificity and lower sensitivity in screening for common disorders, dependent on the balance between the value of early detection and treatment and the costs of false positives [66]. Of course, given lower sensitivities, it is also imperative that results from DPSTs are provided to pediatricians along with guidance on how to think about results accurately, how to convey those results to the family, and what steps to take next.

The use of DPSTs may help mitigate concerns regarding bias introduced by child or caregiver perceptions, since DPSTs rely entirely upon objective data. DPSTs for mental health screening may even detect unobservable mental health biomarkers—like internal hyper-responsiveness to stress—that exhibit especially high discordance between child and caregiver reporters [62]. DPSTs have been used in research settings to detect mental health disorders in children as young as three years old [44] and they raise the possibility that DPSTs could identify children earlier in life before symptoms have the chance to worsen with age [67,68]. The ecological validity of DPSTs must continue to be examined. Emerging evidence suggests that a child’s behavior during laboratory mood induction tasks is representative of their behavior at school and at home (as rated by parents), but additional investigation, that considers a wider range of child emotional and behavioral problems, is needed [69].

While digital phenotyping removes human subjectivity from child mental health screening, it may nonetheless exhibit or introduce new sources of bias. AI tools for mental health are often blind to potential social confounders of the relationship between physical activity or physiological reactivity and mental health states, such as access to community resources or neighborhood safety. Furthermore, young children, females, and patients of color have been underrepresented to date in studies of digital phenotyping for mental health screening, making it impossible to exclude the possibility that human biases have been unwittingly incorporated into the function of current digital phenotyping technologies [16,17,18,70,71,72,73]. To address this concern, there is a need for additional studies examining the sensitivity and specificity of DPSTs in different demographic groups, especially in marginalized communities who might most be harmed by the application of mismatched technology to their health care needs. This will require DPST algorithms to be developed on data from large and diverse user populations.

## 3. Privacy

Through the passive collection of novel data, DPSTs raise new privacy concerns of particular relevance for children. Privacy interests in digital phenotyping for mental health are inherently different in children than for adults [74]. While adults can consent to the collection by DPSTs of their personal biometric data, parental or guardian consent is typically required in the care of minors in most US states [75]. Even when caregivers do consent to data collection for digital phenotyping, it is not clear that they have an adequate appreciation for the associated privacy risks or knowledge of their child’s personal privacy preferences [76].

Most DPSTs involve third-party companies in the diagnostic process [77] and utilize smartphones and wearable devices which produce data accessible to their manufacturers [78]. This poses a risk of unregulated corporate access to data associated with stigmatized pediatric mental health diagnoses and thus rightly raises privacy concerns for patients and families. Moreover, accelerometers—a modality common to many DPSTs—can determine which activities a user is completing, a function which adolescents, in particular, may find troubling especially since third-party commercial companies may be able to access those data [79].

In the US, under the Children’s Online Privacy Protection Act (COPPA), companies are not allowed to knowingly collect personal information from children under the age of 13 years. Both adults and children’s medical records are considered Protected Health Information (PHI) under the Health Insurance Portability and Accountability Act (HIPAA). If a wearable device is used by covered entities in a clinical health setting for adults or children, the data collected, stored, or transmitted are subject to HIPAA regulations. However, HIPAA regulations do not apply to wearables companies because they are not considered covered entities and not always as a business associate to a covered entity [80]. HIPPA compliance is primarily considered the responsibility of the covered entity. HIPAA only extends to the individual and the covered entity (and business associates) and not necessarily the wearable company, raising privacy concerns.

To protect the privacy of pediatric patients undergoing DPST screening for mental health diagnoses, special data protections should be in place prior to use in clinical settings including agreements from all third parties (i.e., wearable device manufacturers) to adhere to HIPAA requirements even if not strictly required. Alternatively, restricting the use of DPSTs to those screening tools that do not communicate a child’s data to third-party wearable companies could also preserve the child’s privacy under COPPA and HIPAA, and avoid the current ambiguity between HIPPA and the PHI being collected. In either case, DPST screening for mental health diagnoses in children should occur in a transparent fashion and following robust parent-informed consent processes and, if feasible, pediatric assent.

To prevent unconsented or inappropriate acquisition of pediatric mental health information by corporate entities, digital phenotypes of mental health should be considered protected health information and thus disclosure of these data and metadata to corporate entities should be covered under HIPAA as a condition of use. As novel devices are developed or commercial devices are utilized for mental health screening purposes, devices and software inherent to DPSTs will need to be developed in regulated environments that are considered covered entities, something the FDA is already exploring [81,82].

**Figure 4:**
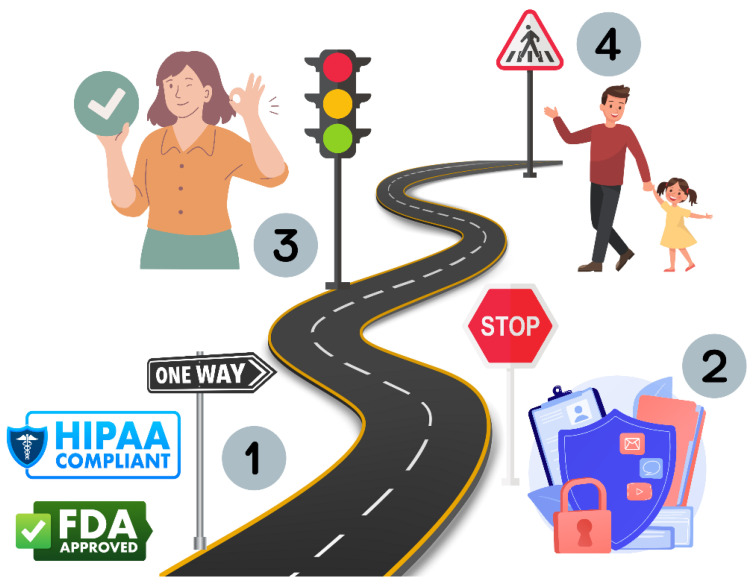
Checklist of privacy standards DPSTs should meet for use in childhood mental health screening. (1) HIPPA-compliant and FDA-approved. (2) Transparent data sharing and storage practices. (3) Caregiver-informed consent. (4) Child assent when reasonably possible.

Beyond the privacy concerns raised by the disclosure of DPST data to third-party companies, there should be clear delineation of which data pediatric patients can access, correct, and revoke. Furthermore, since many states allow children to access mental health care without requiring caregiver consent, children undergoing digital phenotype-based screening should retain the ability to hide their data from their caregivers in accordance with existing local laws. We expect that best practices regarding pediatric privacy protections in the DPST space will evolve in the coming years and continue to require input from lawyers, ethicists, and the community at large. For now, we provide a roadmap of privacy best practices for DPSTs in Figure 4.

## 4. Accessibility and Implementation

Current mental health screening surveys are not widely used in pediatric practice. In a survey of primary care clinicians in 204 practices, 50% of clinicians reported that they never used standardized screening tools to assess the mental health of their pediatric patients [10]. One reason pediatricians report that they do not use such tools is a lack of time: it takes a significant amount of time for pediatricians to administer the surveys, for parents or guardians to complete their portions of screening surveys, and for staff or clinicians to score and interpret survey responses [8]. Pediatricians also report frustration with the lack of mental health providers available for referral, as well as the long waitlists for children to be seen [8]. That is, even if pediatricians dedicate the time to administer screening surveys, children who screen at a high risk for mental health disorders often cannot begin treatment promptly.

**Figure 5:**
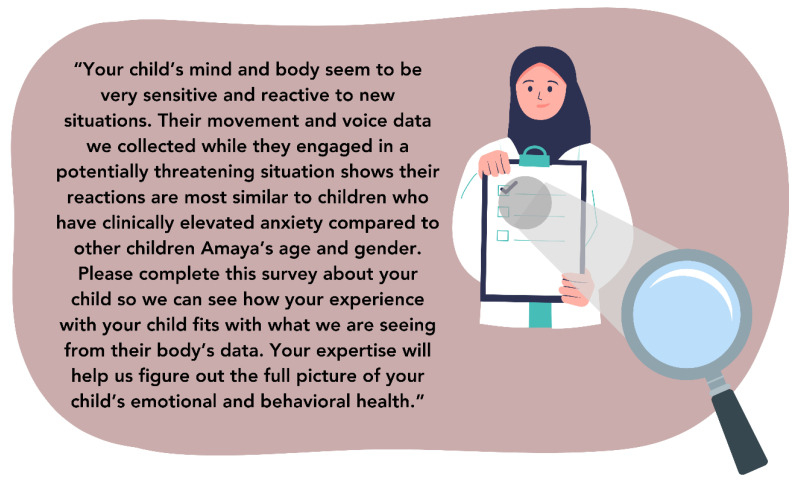
Example patient feedback from DPSTs.

Since screening using DPSTs may take as little as one to three minutes to complete, it may alleviate some of pediatricians’ concerns about time constraints to provide children access to recommended mental health screening [44]. DPSTs could be further configured to save pediatricians time by providing automated clinical notes and therapeutic feedback to families. For instance, feedback from DPSTs to pediatricians could be formatted to pass directly to patient families, provide its own clinical decision-making language [83], and support screening with additional caregiver and pediatrician involvement as necessary. An example of what this note could look like for our example patient, Amaya, is below in Figure 5. Further testing of DPSTs in conjunction with caregiver-reports could help guide screening workflow recommendations. For example, these investigations could indicate if simultaneous or sequential ordering of screening tools yields the best screening performance.

To be successful, DPSTs likely require front-end staff training to ensure standardized instrumentation and task administration. To that end, assessing the feasibility of device placement and task administration within the context of use should be front of mind for DPST developers in their earliest stages.

While DPSTs may help surmount time constraint-related obstacles to recommended mental health screening in children, DPSTs may not directly address the current dearth of mental health providers for children. Rather, if DPSTs fuel a surge in mental health referrals, pediatric access to mental health care could worsen unless there is an increase in the availability of mental health care services, or a reduction in the need for pediatric mental health care. The emergence and scaling of virtual and digital mental health services beginning during the COVID pandemic may help to partially address previously unmet pediatric mental health care needs. Additionally, earlier identification of mental health needs made possible by DPSTs may not always require 1:1 care by a mental health provider. Intervention in early childhood often involves psychoeducation, relational aspects, and teaching of coping strategies to the child’s caregivers [84,85], who are, in turn, capable of supporting the child and helping them learn and implement those strategies in their everyday lives. In early childhood, recommendations for bibliotherapy [86,87] and online programs [88] can be effective and may be sufficient for mitigating current and future mental health risk. The benefit of early identification, when brain plasticity is highest [89], is that small adjustments in everyday life can make a substantial impact. Moreover, receiving a mental health diagnosis, even without treatment, has been shown to be validating and helpful if delivered thoughtfully [90]. In this context, caregivers can be active participants in deciding when the child may need care in the future and have a better idea of why their child may be feeling and behaving the way that they are.

Upfront cost, too, can influence equitable access to DPSTs. The wearable devices and smartphones utilized as DPSTs often cost hundreds of dollars and thus are far more expensive than current screening surveys like the SCARED and PHQ-9 for adolescents (which can be freely accessed online). Yet, the larger upfront costs of DPSTs may be at least somewhat offset by reduced costs of administration and interpretation in pediatric offices. Some devices currently used in research are reusable between patients [21] and can be purchased and lent out by hospital systems under a pay-per-use business model, whereas others may be single-use [91] and have associated costs for the patient (i.e., sensor adhesives or patient/information technology (IT) time trouble shooting). Research on the usability of lower-cost wearable devices in digital phenotype-based screening may therefore be critical to the viability of the widespread use of DPSTs.

Meaningful accessibility of DPSTs will be dependent not only upon pediatricians’ acceptance of the tools, but on children’s acceptance of the tools as well. Young people, who trust digital mental health information more than their elders [92], may feel more comfortable being screened for mental health disorders via DPSTs, rather than by survey. Additionally, like biofeedback therapeutic interventions (which some researchers suggest carry less stigma than traditional psychological treatment [93]), DPSTs measure physiological parameters rather than more subjective personal information [94] and thus patients may feel is less sensitive or stigmatized. This may be of particular importance in African American and Latino ethnic populations who may be more likely to value mind–body connection in mental health care [95,96].

## 5. Limitations

Our aim in this paper has been to enumerate some of the ethical promises and challenges of DPSTs under three broad categories (accuracy and bias, privacy, and accessibility and implementation), particularly in comparison to the screening surveys used sometimes in clinical practice. This is not an exhaustive analysis of all of the possible ethical dimensions of DPSTs. Some topics such as the risk of stigma from mental health diagnosis made by DPSTs warrant additional discussion after some of the more foundational and technology-specific issues taken up here are resolved. 

Given our focus on DPSTs, specifically, we have considered only in passing the more general question of whether young children should be diagnosed with mental health disorders, particularly with a current shortage of treatment options.

Additionally, we have considered the use of DPSTs by pediatricians, as aligned to recommendations of the USPSTF and the AAP Task Force on Mental Health. DPSTs could be used without pediatrician involvement, such as in schools or at home (with or without caregiver supervision). We imagine school use begets similar ethical issues as pediatric use, perhaps with the added issue of governmental involvement in private mental health issues, and the protections of HIPAA may not be upheld in schools. Were DPSTs to be used at home without involvement of a pediatrician, issues with deployment, result interpretation, and referral to mental health care may be more likely.

## 6. Conclusions

**Figure 6:**
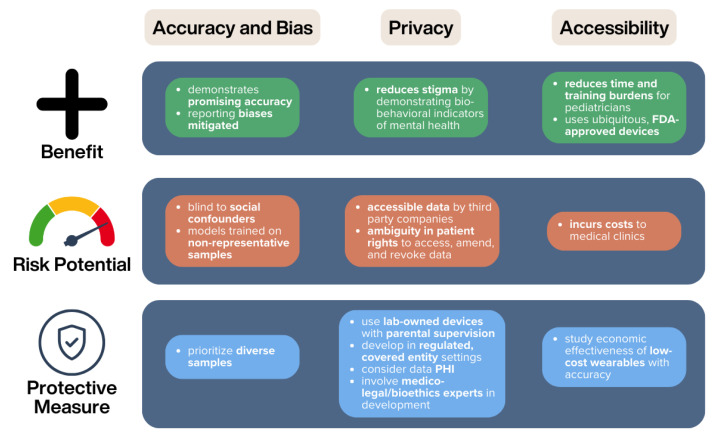
Summary of the ethical risks and benefits of DPSTs and suggested protective measures to enable appropriate clinical use.

In this paper, we identify ethical promises and challenges of DPSTs related to accuracy and bias, privacy, and accessibility and implementation. We have found that multiple ethical challenges remain unsolved when it comes to DPSTs, but these may be ameliorated by the concrete measures suggested in Figure 6. To address some of these challenges, future research should focus on ensuring that affordable DPSTs are developed and evaluated on representative patient samples, with robust corporate privacy protections. Despite these challenges, we believe that DPSTs show significant promise for use in pediatric practice. They have demonstrated accuracy, eliminate concerns regarding under- and over- reporting, and may help to destigmatize mental health problems.

## Data Availability

No new data were created as part of this paper.
